# A Statistical Concept for Conditional Marketing Authorisation Based on the Intermediate and Final Outcomes of a Single Confirmatory Randomised Clinical Trial

**DOI:** 10.1002/pst.70078

**Published:** 2026-02-17

**Authors:** Xiaofei Liu, Nele Henrike Thomas, Elina Asikanius, Caroline Pothet, Anika Großhennig, Armin Koch

**Affiliations:** ^1^ Institute for Biostatistics Hannover Medical School Hannover Germany; ^2^ Biostatistics and Special Pharmacokinetics Unit Federal Institute for Drugs and Medical Devices (BfArM) Bonn Germany; ^3^ Finnish Medicines Agency (Fimea) Turku Finland; ^4^ European Medicines Agency (EMA) Amsterdam the Netherlands

**Keywords:** clinical trials, conditional marketing authorisation, co‐primary endpoints, decision‐making, dual primary endpoints, group sequential design

## Abstract

Conditional marketing authorisation (CMA) is a path to early market access of new medicines addressing an unmet medical need in the European Union (EU), and similar concepts exist in other regulatory regions. For justifying a CMA, the benefit‐risk ratio has to be positive, and the applicant must be able to provide comprehensive data post‐authorisation for converting the CMA to a full marketing authorisation (MA). A recent proposal is to plan a single randomised clinical trial with interim analyses and base the decision for CMA and full MA on an intermediate and a final primary endpoint. To control the study‐wise type‐1‐error (T1E), the dual primary endpoint concept, essentially a Bonferroni‐split of the study‐wise T1E between the intermediate and final endpoints, has been proposed. We argue that the resulting statistical definition of formal study success is not in line with clinical assessment of the overall trial outcome. Consistent with the European Medicines Agency (EMA) guideline on multiplicity issues in clinical trials, the intermediate and final endpoints should be co‐primary, and we propose that each is assessed with a standard group sequential design. We illustrate our proposal using an oncology phase III trial with complete remission (CR) as the intermediate and overall survival (OS) as the final primary endpoint. Based on the example, we demonstrate that our proposal is a valid and flexible alternative with minimal to no impact on costs in terms of sample size or timing of the interim analysis intended for applying for a CMA but it improves the interpretation of the overall trial outcome.

## 1 Introduction

European regulation allows the so‐called conditional marketing authorisation (CMA) as a path to early market access of new medicines if they are intended for treating, preventing or diagnosing seriously debilitating or life‐threatening diseases, including orphan medicines. The settings where this is possible are restricted to situations where all of the following conditions are fulfilled: (i) the benefit‐risk balance of the medicine is positive; (ii) it is likely that the applicant will be able to provide comprehensive data post‐authorisation; (iii) the medicine fulfils an unmet medical need and (iv) the benefit of the medicine's immediate availability to patients is greater than the risk inherent in the fact that additional data are still required [1]. Specifically, the applicant must submit data to substantiate that the benefit‐risk ratio is positive and define conditions (called ‘specific obligations’) that can likely be fulfilled within defined timelines post‐authorisation (i.e., provide additional data) to justify the conversion of the CMA into a standard/full marketing authorisation (MA).

Multiple development strategies for obtaining a CMA exist and, similarly, various approaches can be envisaged as specific obligations to be fulfilled for granting a full MA. In some instances, a CMA was based on the results of a single‐arm clinical trial, which was transformed into a full MA after assessing the results of a randomised clinical trial conducted post‐authorisation. The two trials usually have different primary endpoints, for example, overall response rate (ORR) and overall survival (OS) in the field of oncology.

The downsides of this approach became obvious over time: As soon as a new medicine is available on the market, randomisation to control (placebo, the standard of care or an alternative treatment) in precisely the same indication becomes extremely challenging. The CMA is only an option if an ‘unmet medical need’ under serious and life‐threatening conditions is undisputed. The mere availability of the new medicine on the market is perceived as signalling that sufficient information is available to justify early market access and consequently the proven benefit of the new medicine compared to the control proposed for randomisation. In a non‐negligible number of cases, it became difficult or even impossible to fulfil the condition of providing comprehensive data in a reasonable timeframe [2].

An elegant approach to avoid these difficulties is the recent proposal from several applicants to plan a single randomised trial and base the CMA and final decision about a full MA on its results. In the U.S. Food and Drug Administration (FDA) initiated project FrontRunner [3], the ‘one trial approach’ has been extensively discussed and some methodological proposals have been made which may fit the setting of accelerated approval [4, 5]. In the EU, completing ongoing studies is indeed mentioned in the legislation as one option to define (and fulfil) a specific obligation [1]. Assessment of one outcome variable (we refer to this outcome variable as *intermediate endpoint*) at an interim analysis may justify the CMA, and a subsequent (final) analysis of another outcome variable (we refer to this outcome variable as *final endpoint*) will then support the full MA. The respective regulation states ‘The holder should be required to complete or initiate certain studies with a view to confirming that the risk‐benefit balance is positive and resolving any questions relating to the quality, safety and efficacy of the product’ [1]. Thus, in some instances, it may suffice to confirm that the benefit‐risk balance remains positive based on the specific obligation. Basing the decision about CMA and full MA on the same randomised trial is particularly well suited if questions regarding efficacy remain. It ensures that after completion of the trial an assessment of efficacy as well as the positive benefit‐risk balance according to the usual standards is possible to convert the CMA to a full MA. As an example, consider the field of oncology, where the decision for a CMA has been based on ORR in a single‐arm trial. The agreed specific obligation would be a randomised clinical trial post‐authorisation investigating OS. Compared to this ‘traditional’ way, basing the decision about conditional and full MA on the results of a single randomised clinical trial offers additional advantages. The interim analysis of the intermediate endpoint (ORR) can be carefully positioned in the timeline of the trial while optimising the amount of information available for assessment. However, it is equally important to design the study in a way that its integrity is not compromised by the interim analysis of the intermediate endpoint (and the process of granting CMA), which may jeopardise the further conduct of the trial if, for example, the knowledge about the CMA decision impacts further recruitment of patients, patient retention, or the assessment of a (subjective) outcome variable.

In the following, we will illustrate how the proposal of basing the decision about CMA and full MA on the same trial can be correctly incorporated into the study plan and explain why the often‐applied dual primary endpoint concept [essentially an (uneven) Bonferroni‐split of the study‐wise type‐1‐error (T1E) between the intermediate and final endpoints] is not in line with the clinical assessment of the overall trial outcome. Instead, we argue that defining co‐primary endpoints, each assessed with a standard group sequential design (GSD) testing procedure, as appropriate, is a suitable strategy to align statistical and regulatory decision‐making already at the planning stage of a clinical trial with minimal to no impact on costs in terms of sample size and trial duration.

## 2 Planning Considerations

With the dual primary endpoint concept (also known as ‘alternative primary endpoints’, the ‘at‐least‐one‐approach’ or a ‘primary endpoint family’) [6, 7, 8], which is essentially a Bonferroni‐split of the study‐wise T1E between the primary endpoints, formal study success can be concluded as soon as at least one of two (or more) primary endpoints is statistically significant (and the overall benefit‐risk balance is considered positive). Although the probability of a false‐positive conclusion from the trial is correctly controlled from a statistical perspective, formal study success can be concluded based on a beneficial effect in one primary endpoint even if no effect or (in extreme cases) a (small) detriment in the other primary endpoint is observed. It has been discussed elsewhere that the dual primary endpoint concept is obviously not in line with clinical decision‐making [9].

In the context of CMA, the dual primary endpoint concept has been applied by splitting the study‐wise T1E between the intermediate and final endpoints. The likely intent was to base the decision about CMA on a formally significant effect in the intermediate endpoint. Although this is appreciated, it is not in line with regulatory decision‐making. If questions on efficacy need to be resolved with the specific obligation, the main objective remains to demonstrate efficacy for the final endpoint as a basis for concluding formal study success (and an overall positive trial outcome). As explained above, this is obviously not covered by the dual primary endpoint concept.

Even if a correlation or surrogacy of the intermediate endpoint with/for the final endpoint is plausible, significance of the final endpoint is not guaranteed [10]. Likewise, the benefit‐risk balance may be considered overall positive when the intermediate endpoint is analysed. However, the mere fact that a MA is granted as conditional indicates that additional information for formal proof of efficacy is considered necessary for appropriate decision‐making. Consequently, and in line with the EMA guideline on multiplicity issues in clinical trials [11], the intermediate and final endpoints should be defined as co‐primary (i.e., both endpoints need to be positive for the study to be a formal success).

In recent proposals, the interim analysis of a trial was the only and thus the final analysis for the intermediate endpoint whereas the remaining T1E was spent for the final endpoint. One may argue that the co‐primary endpoint approach has the disadvantage that the trial cannot proceed or will be considered formally unsuccessful if the intermediate endpoint is not positive at the interim analysis intended for a CMA. In contrast, with the dual primary endpoint approach, the trial may still have a second chance of being formally successful if the intermediate endpoint is not positive at the interim analysis, but the final endpoint is positive at the end of the trial. Moreover, with dual primary endpoints, an efficient use of the T1E can be achieved by recycling the T1E allocated to the intermediate endpoint to the assessment of the final endpoint as soon as statistical significance has been demonstrated for the intermediate endpoint, for example in a graphical procedure [12]. Despite these advantages, the dual primary endpoint concept does not fulfil the requirement for a rigorous approach to support proper clinical decision‐making.

Considering all the aforementioned issues of the so far discussed approaches, we propose an alternative strategy for obtaining a CMA which examines the intermediate and final endpoints as co‐primary endpoints, each assessed using a standard GSD testing procedure [13]. With this strategy, the advantage of the dual primary endpoint approach can be retained, that is the study can proceed to the final analysis of the final endpoint even if the intermediate endpoint does not achieve a statistical significance at the interim analysis. At the same time, statistical and clinical conclusions regarding formal study success are aligned before entering the discussion about the benefits and risks of the experimental treatment under investigation. Generally, the interim and final analyses of the intermediate and final endpoints should be positioned at the same time‐points, since at the time of CMA not only a significant treatment effect for the intermediate endpoint should be demonstrated, but also an adequate amount of data for the final endpoint may need to be provided for the benefit‐risk assessment (e.g., to ensure that there is at least no detriment in OS). Formally, this can be implemented by incorporating a non‐inferiority (NI) test with a pre‐specified NI margin for the final endpoint at the interim analysis using the group sequential closed (GSC) test procedures introduced in Wang et al. (2001) [14].

Additional flexibility can be achieved by planning the interim analysis with different information fractions for the two co‐primary endpoints using two separate alpha‐spending functions, each spending the full alpha up to the common final analysis. The intermediate and final endpoints can then be tested at the interim and final analyses with different adjusted T1Es:

- If both the intermediate and final endpoints are significant at the interim analysis, the trial is already formally successful at the interim analysis, and a full MA may be granted directly if the overall benefit‐risk ratio is considered positive.
- If only the intermediate endpoint is significant at the interim analysis, and there is no detriment in the final endpoint (or a positive trend can be shown), a CMA may be justified and the trial will proceed to the final analysis. In case the final endpoint is significant in the final analysis, the trial is formally successful, and the CMA may be converted into a full MA assuming there are no other findings questioning that a positive benefit‐risk ratio remains.
- If the intermediate endpoint is not significant at the interim analysis, the trial can proceed to the final analysis. If both the intermediate and final endpoints are significant at the final analysis, the trial is formally successful from a statistical perspective to support a full MA (again, assuming the benefit‐risk ratio is overall positive).

Certainly, in both the co‐primary and dual primary endpoint approaches, it is possible that the intermediate endpoint does not show a significant treatment effect in the final analysis, while only the final endpoint is significant at the end of the trial. Even if this substantially contradicts the a priori assumption of correlation or surrogacy of the intermediate endpoint with/for the final endpoint, the trial may still be a candidate for an in‐depth benefit‐risk assessment as a basis for the decision about a full MA. However, this can only be achieved if the inconsistencies between the intermediate and final endpoints can be sufficiently explained and do not put the overall decision about a positive benefit‐risk ratio into question.

Finally, an in‐depth assessment of benefits and risks may likewise be accepted to convert the granted CMA into a full MA if the final endpoint is not superior at the end of the trial. Here, a positive trend in the final endpoint may be sufficient for converting it into a full MA. Therefore, we strongly recommend pre‐defining a tight NI margin already at the planning stage of the trial to ensure the expected positive trend. However, if neither superiority nor NI with the pre‐specified margin can be demonstrated for the final endpoint at the final analysis, the trial is deemed formally unsuccessful. Table 1 summarises the definition of formal study success based on dual and co‐primary intermediate and final endpoints.

**TABLE 1:** Definition of formal study success based on dual and co‐primary intermediate and final endpoints before benefit‐risk assessment for CMA and/or full MA.

Dual primary endpoints					
IA		FA		Conclusion on study success	Candidate (or eligible) for benefit‐risk assessment for CMA and/or full MA
IEP	FEP	IEP	FEP		
✓	✓	—	—	Formally successful	Yes for full MA (at the IA).
✓	✗	—	✓	Formally successful	Yes for both CMA (at the IA) and full MA (at the FA).
✓	✗	—	✗	Formally successful	Yes for CMA (at the IA), but specific obligation for full MA (at the FA) not fulfilled.
✗	✓	—	—	Formally successful	Yes for full MA (at the FA).
✗	✗	—	✓	Formally successful	No for CMA, but yes for full MA (at the FA).

Co‐primary endpoints					
IA		FA		Conclusion on study success	Candidate (or eligible) for benefit‐risk assessment for CMA and/or full MA
IEP	FEP	IEP	FEP		
✓	✓	—	—	Formally successful	Yes for full MA (at the IA).
✓	✗	—	✓	Formally successful	Yes for both CMA (at the IA) and full MA (at the FA).
✓	✗	—	✗	Formally not successful	Yes for CMA (at the IA), but specific obligation for full MA (at the FA) not fulfilled.
✗	—	✓	✓	Formally successful	No for CMA, but yes for full MA (at the FA).
✗	—	✗	✓	Formally not successful	An in‐depth discussion of a trial which failed to demonstrate one of its primary objectives may in instances be warranted.

Abbreviations: ✓: significant; ✗: not significant; CMA: conditional marketing authorisation; FA: final analysis; FEP: final endpoint; IA: interim analysis; IEP: intermediate endpoint; MA: marketing authorisation.

## 3 Example

A randomised controlled pivotal phase III oncology clinical trial is planned to demonstrate the superiority of a new therapy (N) as compared to a standard therapy (S) for the marketing authorisation application (MAA) in the intended indication. Dual primary endpoints with complete remission (CR) as the intermediate endpoint and OS as the final endpoint are proposed initially. A CMA based on a significant effect on CR is intended and a conversion into a full MA is foreseen if a significant effect in OS is shown. To strongly control the study‐wise T1E at 0.025 (one‐sided), an asymmetric Bonferroni‐split of the full alpha between the two primary endpoints is proposed, where a one‐sided alpha of 0.002 is allocated to CR and 0.023 to OS. In total, two analyses at two time‐points during the trial are planned, where the interim and final analyses will assess both CR and OS, respectively. The Lan‐DeMets (O'Brien‐Fleming‐type) spending function is utilised to determine the efficacy boundaries [15]. An improvement in the CR rate from 36% (S) to 55% (N) and a prolongation of the median OS time from 15 months (S) to 22.7 months (N) are assumed for the sample size calculation. With a 1:1 randomisation ratio, a uniform recruitment period of 17 months, a minimum follow‐up time of 11 months per patient and exponentially distributed survival times, a total of 496 patients and 254 death events will provide at least 92% and 89% power for CR and OS, respectively. The pre‐specified analyses, respective time‐points and information fractions for dual intermediate and final primary endpoints are summarised in Table 2.

**TABLE 2:** Study plan based on dual intermediate and final primary endpoints.

Analysis	Time‐point	Information fraction Adjusted one‐sided alpha Power	
		CR (alpha = 0.002)	OS (alpha = 0.023)
IA	194 death events occur or 6 months after completion of randomisation (whichever occurs later) (about 23 months after FPFV)	496 patients (100%) alpha = 0.002 power = 92%	194 events (76%) alpha = 0.00916 power = 70%
FA	254 death events occur (about 28 months after FPFV)	—	254 events (100%) alpha = 0.02027 power = 89%

*Note:* The adjusted one‐sided alphas were calculated using the SEQDESIGN procedure in SAS 9.4 and the ceiling‐adjusted design boundary information was utilised.

Abbreviations: CR: complete remission; FA: final analysis; FPFV: first patient first visit; IA: interim analysis; OS: overall survival.

To align statistical and clinical criteria for formal study success regarding the primary endpoints, we propose considering CR and OS as co‐primary endpoints. In this situation, each of them can be tested at the one‐sided alpha level of 0.025. One interim and one final analysis for both CR and OS at common time‐points are planned. The Lan‐DeMets (O'Brien‐Fleming‐type) spending function is utilised to determine the efficacy boundaries. The pre‐specified analyses, respective time‐points, information fractions and adjusted one‐sided alphas for co‐primary intermediate and final endpoints are summarised in Table 3. Primarily, we intended to maintain the overall assumptions (i.e., expected treatment effects on both endpoints, length of recruitment and follow‐up period and number of patients/events) and the decision criteria for applying a CMA (i.e., CR should be significant at the one‐sided alpha of 0.002 at the interim analysis) of the original plan. Therefore, the information fractions for CR and OS at the common interim analysis were adapted, so that the alpha allocated to CR in the interim analysis is as similar as possible to the alpha for CR in the original plan (i.e., one‐sided alpha of 0.002).

**TABLE 3:** Study plan based on co‐primary intermediate and final endpoints (O'Brien‐Fleming‐type spending function).

Analysis	Time‐point	Information fraction Adjusted one‐sided alpha Power	
		CR (alpha = 0.025)	OS (alpha = 0.025)
IA	About 15 months after FPFV (Recruitment completion: 88%)	254 patients (53%) completed 6 months alpha = 0.00211 power = 57%	100 events (41%) alpha = 0.0004625 power = 11%
FA	About 28 months after FPFV	478 patients (100%) alpha = 0.02432 power = 99%	245 events (100%) alpha = 0.02484 power = 90%

*Note:* The adjusted one‐sided alphas were calculated using the SEQDESIGN procedure in SAS 9.4 and the ceiling‐adjusted design boundary information was utilised.

Abbreviations: CR: complete remission; FA: final analysis; FPFV: first patient first visit; IA: interim analysis; OS: overall survival.

As can be seen in Table 3, the revised design proposal neither leads to an increase in the required number of patients, nor to a delay in the timeline for the planned CMA and full MA, as compared to its counterpart using the dual primary endpoint approach. As the information fraction for CR in the interim analysis is reduced to about 53%, correspondingly, the interim analysis can be conducted at an earlier time‐point than in the original plan with reduced power. If statistical significance cannot be achieved at the interim analysis, there is still a great chance (i.e., sufficient power) for CR at the final analysis, since with the co‐primary endpoint concept, each endpoint can be tested at the full alpha of 0.025. With co‐primary endpoints it is usually recommended to increase the power for each endpoint to 90% to achieve an overall power of 80%. While it may still be wise to increase the power for the assessment of OS beyond 80%, this may not be strictly needed because the number of patients in the study is completely driven by the final endpoint OS, and the intermediate endpoint is substantially overpowered in most instances. For example, the study planned with 90% power for OS has a power of 99% for the assessment of CR at the final analysis.

An alternative proposal is to use a Hwang‐Shih‐DeCani spending function for CR [16]. By setting different values for the gamma parameter of the spending function, different information fractions for CR at the interim analysis can be considered, while the allocated alpha to CR in the interim analysis remains similar to that in the original plan (i.e., one‐sided alpha of 0.002). In the alternative proposal presented in Table 4, the gamma parameter was set to −21. For OS the O'Brien‐Fleming‐type spending function was still applied. The Hwang‐Shih‐DeCani spending function enables utilising more information in the interim analysis as compared to the O'Brien‐Fleming‐type spending function, in case a higher power for testing CR at the interim analysis is desired. As the information fraction for CR in the interim analysis increases, correspondingly, the interim analysis will be conducted at a later time‐point than in the study plan presented in Table 3.

**TABLE 4:** Study plan based on co‐primary intermediate (Hwang‐Shih‐DeCani spending function) and final (O'Brien‐Fleming‐type spending function) endpoints.

Analysis	Time‐point	Information fraction Adjusted one‐sided alpha Power	
		CR (alpha = 0.025)	OS (alpha = 0.025)
IA	About 21 months after FPFV (Recruitment completion: 100%)	426 patients (88%) completed 6 months alpha = 0.00202 power = 86%	178 events (72%) alpha = 0.00805 power = 64%
FA	About 28 months after FPFV	484 patients (100%) alpha = 0.02499 power = 99%	248 events (100%) alpha = 0.02257 power = 90%

*Note:* The adjusted one‐sided alphas were calculated using the SEQDESIGN procedure in SAS 9.4 and the ceiling‐adjusted design boundary information was utilised.

Abbreviations: CR: complete remission; FA: final analysis; FPFV: first patient first visit; IA: interim analysis; OS: overall survival.

The study plan depicted in Table 4 can be supplemented by explicitly pre‐specifying the expected evidence level of the final endpoint at the interim analysis through a tight NI margin (reflecting which degree of inferiority/detriment can be excluded or which positive trend can be shown) based on the planning assumptions.

## 4 Discussion

In this paper, we discussed various approaches that have been proposed for a CMA so far. The dual primary endpoint approach, which splits the study‐wise T1E between the intermediate endpoint (supporting the CMA) and the final endpoint (supporting the full MA), does have some advantages: (i) the Bonferroni‐split of the T1E between the intermediate and final endpoints is statistically correct and easy to implement, (ii) the study is formally successful as soon as the null hypothesis regarding the intermediate endpoint is rejected as a basis for a positive decision about CMA, (iii) an efficient use of the T1E can be achieved by propagating the T1E allocated to the intermediate endpoint to the assessment of the final endpoint as soon as statistical significance can be shown for the intermediate endpoint, (iv) despite a negative result of the intermediate endpoint, the study may still have a chance to be formally successful based on a positive result of the final endpoint at the end and to be considered for granting a full MA.

Despite its several advantages, the dual primary endpoint approach is not in line with clinical decision‐making because this cannot ignore (potential) negative findings in the final endpoint even if a significant finding in the intermediate endpoint is observed [9]. As long as uncertainties remain on efficacy, the specific obligation for the full MA should include a demonstration of efficacy based on the final endpoint. Consequently, the intermediate and final endpoints should be considered co‐primary. Here, formal proof of efficacy on the final endpoint is a prerequisite for concluding formal trial success, which, in combination with a consistently significant positive effect on the intermediate endpoint and a positive benefit‐risk ratio, qualifies the trial for granting a full MA. If the intermediate endpoint is not significant, a trial with a formal proof of efficacy of the final endpoint may still be eligible for a benefit‐risk assessment for full MA, as far as the inconsistencies between the intermediate and final endpoints can be sufficiently explained and regarded to have no negative impacts on the efficacy and safety profile.

We illustrated that the approach to consider the intermediate and final endpoints as co‐primary, assessing each endpoint using a standard GSD testing procedure [13], is a valid and flexible strategy for basing the decision on CMA and the subsequent full MA on the same clinical trial. By carefully choosing GSDs for the intermediate and final endpoints, and specifically the respective timing/information fraction of the interim analysis, this strategy would neither lead to an increase in the overall sample size, nor to a delay in the timing of the CMA, as compared to its counterpart using the dual primary endpoint approach. As above‐mentioned, choosing an appropriate time‐point for performing the interim analysis alone can be challenging and requires consideration of several aspects, for example disease area and chosen primary endpoints. Generally, the timing of the interim analysis may be driven by the requirement that recruitment is almost completed and sufficient information for the final endpoint is available to enable a preliminary benefit‐risk assessment.

Certainly, one may plan to conduct the interim analysis when ‘all’ information is available for the intermediate endpoint (i.e., there will be no increment in the information for the intermediate endpoint until the final analysis) and spend the full alpha on the intermediate endpoint at the interim analysis. This assumes that the treatment effect on the intermediate endpoint or the correlation between the intermediate and final endpoints has not been overestimated (e.g., there will be no late events for time‐to‐event intermediate endpoint). On the one hand, discarding a GSD testing procedure for the intermediate endpoint can maximise the power for demonstrating superiority in the intermediate endpoint and, consequently, increase the chance for obtaining a CMA. On the other hand, this bears the risk that if the intermediate endpoint is not significant at the full alpha at the interim analysis, the study will already be deemed formally unsuccessful and should theoretically not proceed to the final analysis (i.e., no chance for applying for a full MA), irrespective of the potential results of the final analysis. Therefore, incorporating a GSD testing procedure for the intermediate endpoint into the co‐primary endpoint concept provides an opportunity to balance the chance for granting a CMA and a full MA when the correlation between the intermediate and final outcomes is still speculative. Table 5 summarises the advantages and disadvantages of both dual and co‐primary endpoint approaches.

**TABLE 5:** Pros and cons of the dual and co‐primary endpoint approaches.

	Pros	Cons
Dual primary endpoints	T1E controlled. Simple application. Alpha can be propagated between endpoints. If the claimed surrogacy of the IEP does not hold, the study can still be formally successful based on the final endpoint.	Not in line with clinical decision‐making (the study may be formally successful, even if the FEP is not significant or negative).
Co‐primary endpoints	T1E controlled. In line with clinical decision‐making. Flexible definition of requirements for formal study success. Both the IEP and FEP can be tested at full alpha. Overestimated correlation between the IEP and FEP may result in no significance at the IA but still allow significance at the FA. Study success mandates meeting the specific obligation that is significance in the clinically relevant FEP (from a CHMP position).	If the claimed surrogacy of the IEP does not hold, the study may be formally unsuccessful. Challenging for some indications (e.g., MASH), where time between the IA and FA (until sufficient data for the FEP) is long.

Abbreviations: CHMP: Committee for medicinal products for human use; FA: final analysis; FEP: final endpoint; IA: interim analysis; IEP: intermediate endpoint; MASH: metabolic dysfunction‐associated steatohepatitis; T1E: type I error.

As explained, basing decision‐making for CMA and full MA on the same trial has major advantages. However, the major caveat with such approaches is the risk of damaging the study integrity as a consequence of the interim analysis of the intermediate endpoint. As this analysis is intended for regulatory submission, it means that the study will be fully analysed and reported and hence, the results are available to the sponsor, the investigators, the scientific community and the patients in the trial. This may introduce bias into the study which is difficult to accurately quantify and may impact the further conduct of the study and the evaluation of the final endpoint which is essential for the full MA.

For double‐blind studies, the blind could still be maintained at the patient and investigator levels. Two measures are consequently needed to protect study integrity and to ensure the study completion: Firstly, the interim analysis on the intermediate endpoint should be planned sufficiently late such that the enrolment is at least nearly completed. Secondly, the study should be double‐blind and diligence needs to be exercised to ensure that blinding of patients and investigators is maintained after the interim analysis has been conducted and results have been communicated. An independent data monitoring committee (DMC) should be tasked to perform the interim analysis and only if the conditions for CMA are fulfilled, the conduct of the full analysis in preparation for the application for CMA should then be carried out by the sponsor. This initial analysis of the DMC should preferably also include an evaluation of a positive benefit‐risk ratio before recommending the initiation of the CMA procedure.

Admittedly, blinding is not possible in some instances due to profound differences in treatment regimens. It is difficult to foresee how unbiased assessment can be ensured after group‐level information has unavoidably been made public. In these situations, the proposed strategy may not be worth the additional risk, as the trial would have to be designed to allow very little time between the interim and the final analysis. Consequently, the gain in time to market access would be negligible if the interim analysis is planned at a time‐point where publication of interim findings would not impact the further trial conduct or assessment.

Although we mainly illustrated our proposal using the example from oncology, the proposed approach may also be applied to other non‐oncological indications. Furthermore, indications exist where the proposed approach may not be meaningfully implemented. For example, for metabolic dysfunction‐associated steatohepatitis (MASH), the general procedure for an MA is to base a CMA on acceptable short‐term intermediate endpoints (e.g., histological endpoints) and then a full MA on long‐term final endpoints (e.g., relevant clinical events). As the disease typically progresses slowly, it may take several years for a sufficient number of patients to show progression (i.e., reach pre‐specified clinical events). Therefore, it would be difficult to plan a common interim analysis for the intermediate and final endpoints, where an adequate amount of information for the final endpoint is available without a substantial increase in the sample size or a delay in the timing for CMA. Nevertheless, the operational difficulty does not automatically justify the appropriateness of the dual primary endpoint concept. How to meaningfully implement the co‐primary endpoint concept for such a specific indication deserves further research and discussion.

## Conflicts of Interest

The authors declare no conflicts of interest.

## Data Availability

Data sharing is not applicable to this article as no datasets were generated or analysed during the current study.
